# “Waste Not, Want Not” *—* Leveraging Sewer Systems and Wastewater-Based Epidemiology for Drug Use Trends and Pharmaceutical Monitoring

**DOI:** 10.1007/s13181-021-00853-4

**Published:** 2021-08-16

**Authors:** Timothy B. Erickson, Noriko Endo, Claire Duvallet, Newsha Ghaeli, Kaitlyn Hess, Eric J. Alm, Mariana Matus, Peter R. Chai

**Affiliations:** 1 Department of Emergency Medicine / Division of Toxicology, Brigham & Women’s Hospital / Harvard Medical School, 10 Vining St, Boston, MA 02155 USA; 2 Division of Medical Toxicology, Department of Emergency Medicine, Mass General Brigham, Boston, USA; 3 Harvard Humanitarian Institute, Cambridge, MA USA; 4 Biobot Analytics, Inc., Cambridge, MA USA; 5 Department of Biological Engineering, Massachusetts Institute of Technology, Cambridge, MA USA; 6 Center for Microbiome Informatics and Therapeutics, Massachusetts Institute of Technology, Cambridge, MA USA; 7 Antimicrobial Resistance Interdisciplinary Research Group, Singapore-MIT Alliance for Research and Technology, Singapore, Singapore; 8 Campus for Research Excellence and Technological Enterprise (CREATE), Singapore, Singapore; 9 Broad Institute of MIT and Harvard, Cambridge, MA USA; 10 The Fenway Institute, Boston, MA USA; 11 The Koch Institute for Integrated Cancer Research, Massachusetts Institute of Technology (MIT), Cambridge, MA USA

**Keywords:** Wastewater, Sewers, Surveillance, Opioids, Stimulants

## Abstract

During the current global COVID-19 pandemic and opioid epidemic, wastewater-based epidemiology (WBE) has emerged as a powerful tool for monitoring public health trends by analysis of biomarkers including drugs, chemicals, and pathogens. Wastewater surveillance downstream at wastewater treatment plants provides large-scale population and regional-scale aggregation while upstream surveillance monitors locations at the neighborhood level with more precise geographic analysis. WBE can provide insights into dynamic drug consumption trends as well as environmental and toxicological contaminants. Applications of WBE include monitoring policy changes with cannabinoid legalization, tracking emerging illicit drugs, and early warning systems for potent fentanyl analogues along with the resurging wave of stimulants (e.g., methamphetamine, cocaine). Beyond drug consumption, WBE can also be used to monitor pharmaceuticals and their metabolites, including antidepressants and antipsychotics. In this manuscript, we describe the basic tenets and techniques of WBE, review its current application among drugs of abuse, and propose methods to scale and develop both monitoring and early warning systems with respect to measurement of illicit drugs and pharmaceuticals. We propose new frontiers in toxicological research with wastewater surveillance including assessment of medication assisted treatment of opioid use disorder (e.g., buprenorphine, methadone) in the context of other social burdens like COVID-19 disease.

## Introduction

“The sewer is the conscience of the city. Everything there converges and confronts everything else. In that livid spot there are shades, but there are no longer any secrets.” ~ *Victor Hugo, The Intestine of Leviathan, Les Misérables*

The first documented sewer system was built by the Crete Minoans in 1500 BCE with latrines connected to vertical chutes converging into a common underground area. Ancient Persians, Athenians, Macedonians, and Greeks also designed intricate sewer systems. The Romans built *Cloaca Maxima* the largest sewer of its kind, around 600 BCE. It was only after the mid nineteenth century that that modern sewerage was “reborn,” but many of the principles designed by the ancients are still in use today [1]. In the USA, the first sewer systems were constructed in the 1850s in Chicago and Brooklyn. These systems, consisting of large-bore pipes, collected human liquid and solid waste along with household wastewater. These common sewer lines additionally collected industry runoff as well as rainwater. Given the contamination of sewer systems with industrial chemicals and the propensity to affect overall environmental health, large cities considered the need for wastewater treatment plants to decontaminate and monitor water quality. The first wastewater treatment plant to develop chemical precipitation was in Worcester, Massachusetts, in 1890. Modern-day closed sanitary sewers are composed of elaborate underground pipes or tunnel systems for transporting sewage from houses and buildings to wastewater treatment facilities [2]. Sanitary sewers are part of an overall system called a sewage system or sewerage. Closed municipal sewage systems that collect wastewater from individual houses and commercial buildings include toilet flush consisting of stool and urine, domestic graywater from showers, washing machines, and dishwashers. Modernized systems utilize a dual sewage system draining matrix that separates wastewater from liquid storm runoff. Because individuals deposit liquid and solid waste which includes metabolized and parent compound xenobiotics into toilets, access to wastewater at various points within the sewer systems can provide insights into the population-level drug exposures proximal to these access points.

Historically, epidemiological drug surveillance has relied on clinical overdose information from sources like hospitals, population surveys, and poison center databases. Entry into these public health data repositories relies on individuals notifying or accessing the healthcare system, often well into their disease process. Despite their expansiveness, these systems therefore have inherent biases. With the stigma associated with substance abuse, a large proportion of individuals who use drugs do not present to healthcare systems. This, along with fluctuating testing availability, means that clusters of disease may become widespread before they are identified by traditional health reporting mechanisms. Relying exclusively on clinical statistics, population surveys and poison center data therefore run the risk of underestimating the true scale of a drug use epidemic.

Monitoring and quantifying drug metabolites in wastewater networks permit visualization of clusters of disease regardless of clinical presentations and testing availability. Wastewater-based epidemiology (WBE) leverages existing sewer infrastructures to access aggregated human waste data to inform public health officials and policy makers about population health. Wastewater surveillance can also be integrated into public works and facility-level monitoring programs. Testing wastewater at a regular frequency allows analysis of geographical and temporal trends of drug concentrations to map the evolution of an epidemic in a cost-effective, equitable manner, providing snapshots of disease activity that are independent of individuals’ socioeconomic status or access to healthcare. WBE is a powerful tool to measure dynamics of population-level health by analysis of a variety of biomarkers such as drug metabolites, chemicals, and pathogens in wastewater [3]. WBE has been used as a key technique in surveillance and monitoring of drug use, as well as environmental and toxicological contaminants [4, 5]. Sewage-based measures of specific compounds in wastewater can rapidly establish the presence of pharmaceutical trends in community-wide health [6]. Monitoring of sewage for contaminants, drug metabolites, and compounds formed by sewage interactions can further serve as indicators of human health and longitudinally gauge time trends in population health. Metabolites and agents of interest may be degraded due to high bacterial content but do not generally form with other compounds when mixed with chemicals. On a broad scale, these metabolites are relatively stable and preserved; therefore, additions of sporadic chemicals do not alter larger population analyses.

These techniques have been applied from both epidemiological research and policy perspectives to provide data on community-wide use of tobacco, alcohol, and illicit drugs [7]. Amid the COVID-19 pandemic, renewed interest in using existing wastewater networks to conduct population-level surveillance has galvanized the deployment of many wastewater monitoring systems. In parallel with COVID-19 disease, the resurgence of drugs of abuse may be an important target to monitor. In this manuscript, we describe (1) the basic tenets and techniques of WBE, (2) review its current application among drugs of abuse, and (3) propose methods to scale and develop both monitoring and early warning systems with respect to measurement of illicit drugs and pharmaceuticals.

## Wastewater Sampling Techniques and Resolution

**Fig. 1:**
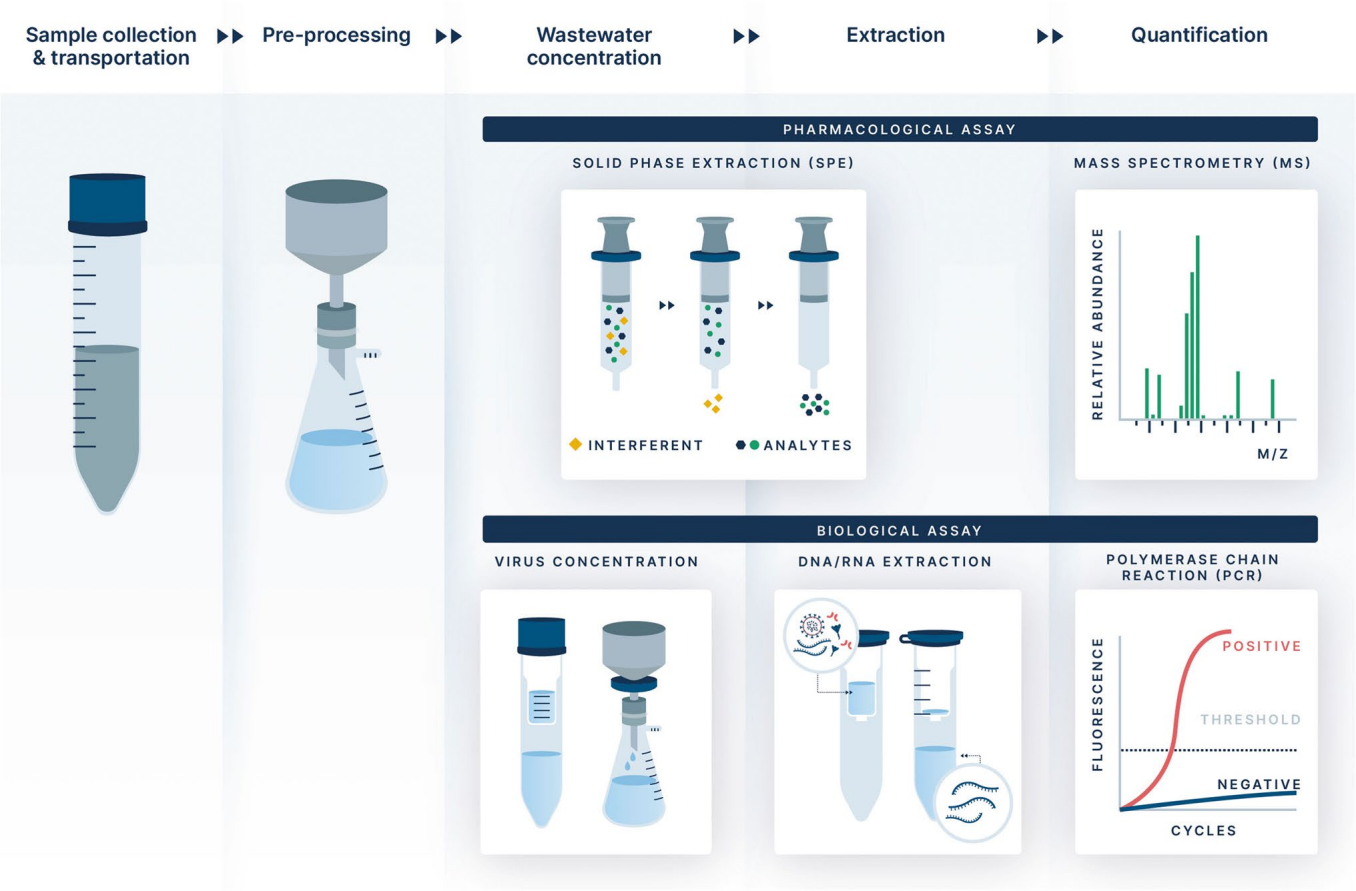
Sampling methods: Solid-phase extraction (SPE) of drugs and pharmaceuticals. Focused polymerase chain reaction (PCR) primers for specific biological agents (Elipse Agency < http://www.elipseagency.com > used with permission)

Operationally, access to the sewer network can be obtained at aggregating sites like wastewater treatment plants (WWTPs), locally at pumping stations, or at the neighborhood level through direct access to manhole portals [8]. Wastewater effluent collected at these sites typically contains both solid and liquid waste. With the use of a solid phase extractor, effluent can be separated, and analysis conducted on specific components of waste to optimize capture of the target analyte or specimen. Both solid and liquid phases can be analyzed with the use of liquid chromatography tandem mass spectroscopy (LC/MS/MS) to measure specific xenobiotics, or polymerase chain reaction (PCR) to measure viral and bacterial fragments (Fig. 1). Once processed, wastewater samples can be stored thereby creating a historical library of human effluent over time.

**Fig. 2:**
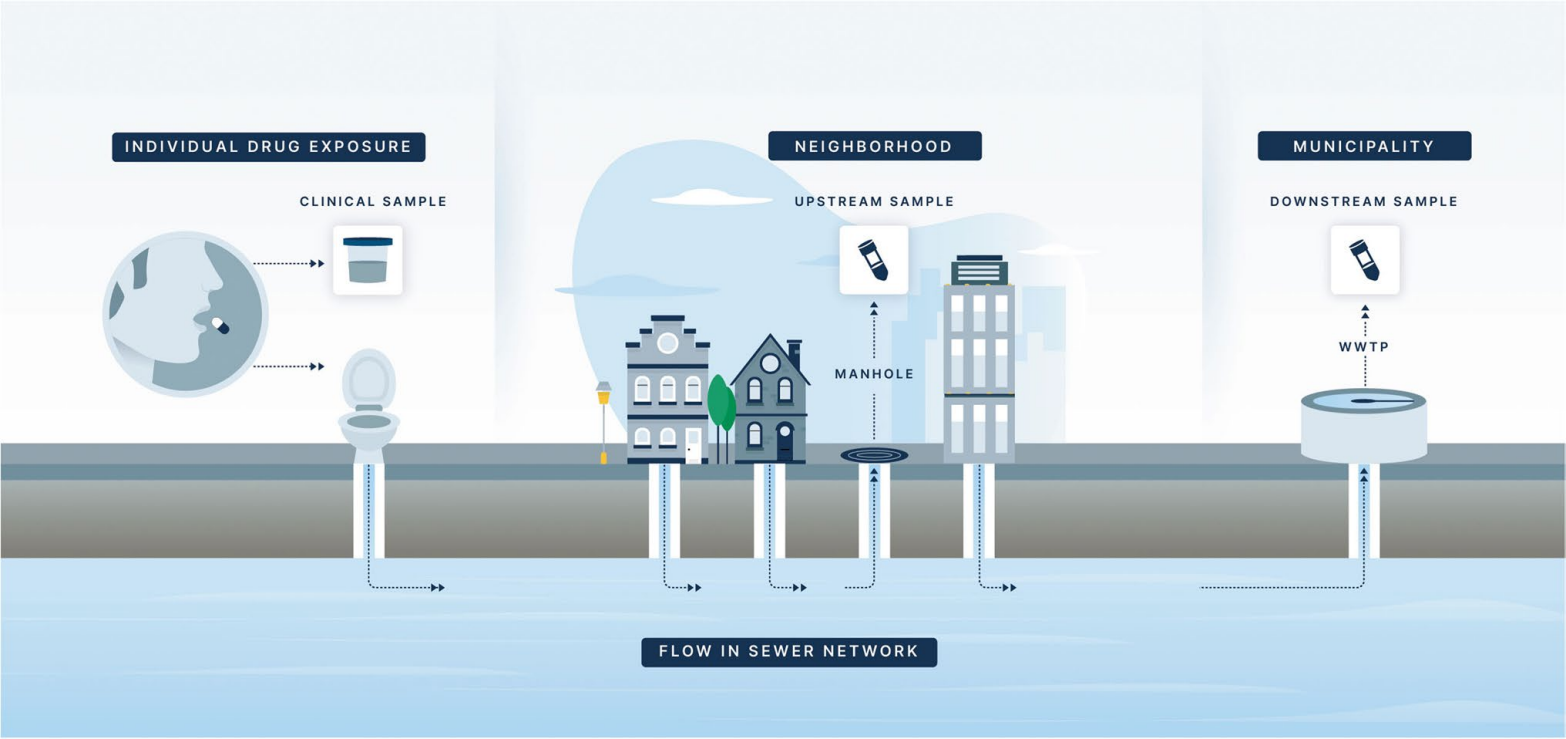
Schematic of a wastewater treatment network with targeted upstream sampling and downstream testing. Analyses of wastewater at various access points within the sewer system can provide insights into the population-level drug exposures within the area (Elipse Agency < http://www.elipseagency.com > used with permission)

While WBE traditionally measures the presence of drugs and their metabolites at the wastewater treatment plant level, varying the source of sample collection allows researchers to adjust the resolution of monitoring. Epidemiologic surveillance can occur downstream at the highest aggregator of sewage (WWTPs) or upstream at pumping stations which serve smaller communities and neighborhoods. While each resolution enables varying types of epidemiological data, downstream and upstream sampling can be combined to complement each other and provide a gross indicator of population health. In this section, we describe techniques to access the sewer network at both downstream and upstream sampling sites (Fig. 2).

### Downstream Sampling

Downstream sampling occurs at wastewater treatment plants or other major endpoints of the wastewater network. Wastewater sampling is a part of regular operations in the USA as the Environmental Protection Agency (EPA) regulates discharge and treatment of wastewater and WWTPs monitor properties of influents and effluents. Because WWTPs are at the most distal point from the origin of waste, downstream analysis provides the maximum level of large-scale population and regional-scale aggregation. Downstream sampling is also easy to accomplish as techniques to sample effluent at the WWTP level are well established. Data from downstream sampling is naturally deidentified and can describe large catchment-level exposures to target analytes. During the COVID-19 pandemic, WWTP-based SARS-CoV-2 surveillance has become a useful adjunct to clinical case reporting in understanding unbiased population-level data around infectious disease exposure [9]. This type of sampling, however, has shortcomings including highly variable waste travel time, and degradation of important human metabolites [10]. Degradation in particular poses challenges for analysis from the toxicological perspective because molecular degradation occurs at different rates due to variable travel time. Therefore, some key indicators may be undetectable at WWTPs.

### Upstream Sampling

In contrast to downstream sampling at WWTPs, surveying multiple locations “upstream,” at the neighborhood level permits detection of geographically sensitive and precise analysis of important drug exposures. Upstream surveillance may facilitate more granular and sentinel detection of catchments with unique toxicological, pharmaceutical, and infectious disease burdens, to enable early warning and inform public health experts regarding at-risk communities [10]. Sampling is accessed from sewerage maintenance portals or manholes where flow aggregates at the scale of thousands to tens of thousands of individuals [8]. In regions with existing infrastructure, it is possible to leverage geographic information system (GIS) analysis to design sampling schemes that include manholes at sentinel locations with specific characteristics such as population level and travel time. To further increase resolution, the wastewater system can be accessed at the level of individual buildings to understand residents’ exposure to specific xenobiotic or biologic agents. This technique has recently been utilized to measure SARS-CoV-2 infections in congregate settings like school dormitories, nursing homes, hospitals, and industrial sites during the COVID-19 pandemic [11]. However, sampling upstream poses additional logistical challenges than sampling downstream as it requires specialized laboratory tools and data interpretation resources. Fluctuations in the contributing population to a sample are more impactful than at the WWTP, requiring the use of normalization markers. Important metadata like daily flow are more difficult to obtain, further limiting which WBE analyses can be implemented. Finally, correlating wastewater-based measurements with an associated population requires knowledge of the underlying sewage network.

Combining downstream and upstream analysis, wastewater sampling can offer real-time, cost-effective surveillance of a community’s health at different levels of population aggregation [10]. In general, only selected parent compounds and metabolites are suitable for downstream WBE given rapid metabolism by the wastewater microbiome. Therefore, upstream sampling is required to capture more unstable metabolites. To account for variation in catchment size, waste travel time, and flow rate, most analysis of key drug metabolites should be correlated with known stable components of wastewater that can serve as internal controls. These components may be pharmacologic (e.g., target analyte standardized to acetaminophen or caffeine) or biologic (e.g., target analyte standardized to pepper mild mottle virus) [12, 13]. By normalizing concentrations of target drugs to these standards, a prevalence estimate can be determined suggesting the degree of exposure to a specific target analyte in the catchment area.

### Infectious Disease Surveillance

In a setting where large-scale in-person testing may not be feasible or is cost-prohibitive, longitudinal analysis of wastewater can provide population-level estimates of infectious disease burden [14]. Recently, WBE has been broadly adopted for COVID-19 surveillance. The Center for Disease Control and Prevention (CDC) established the National Wastewater Surveillance System (NWSS) to harmonize academic and private sector efforts [15]. These efforts detect fragments of virus that are shed by individuals into the wastewater network. By performing reverse transcriptase polymerase chain reaction (RT-PCR), SARS CoV-2 is quantified and viral loads with estimated case prevalence are reported. In parallel to infectious disease surveillance, antiviral drugs have also been detected in wastewater [16], and the consumption of antiviral drugs (e.g., oseltamivir) has been evaluated using sewage epidemiology [17]. These approaches can be extended for additional applications in public health assessment of emerging viral infection outbreaks [3]. WBE represents a complementary approach to clinical surveillance to measure the presence and prevalence of infectious threats such as COVID-19 disease [9, 14]. This innovative data source has improved epidemiological modeling to measure the penetrance of SARS-CoV-2 in communities. It also helps inform decisions surrounding the tailoring of social distancing and quarantine efforts based on dynamic wastewater catchment-level estimations of prevalence [14, 18–20]. WBE can provide early warnings for resurgence [21], and trends for ongoing surveillance to detect emerging infectious disease clusters [6].

### Environmental and Toxicological Contaminants

WBE can be used for the environmental analysis of pesticide biomarkers (triazines, pyrethroids, organochlorines, and organophosphates) as a low-cost complementary biomonitoring tool for assessing population-wide exposure [22]. An expanded WBE pharmacokinetic dataset has been developed describing excretion rates and stability descriptions of 2,4-D, aldrin, carbaryl, chlorobenzilate, dieldrin, diquat, ethion, glufosinate, glyphosate, folpet, malathion, parathion, penconazole, and tebuconazole [23]. There has been a recent development of a sensitive and accurate method for the rapid determination of selected insecticides and herbicides in tap and wastewater samples using vortex-assisted, solvent-based liquid-phase microextraction prior to determination by gas chromatography-mass spectrometry [24]. Despite innovative assay development, there remains a paucity of informational monitoring of these toxicological contaminants.

Like analyzing exposure to drugs and pesticides, WBE can also be applied to the study of environmental heavy metal exposure. The analysis includes biomarkers for various metals (e.g., arsenic, cadmium, cobalt, copper, iron, lead, mercury, nickel, selenium, silver, tin, and zinc) grouped according to toxicological health implications. The use of WBE allows for the interpretation of the relationship between metal exposure (e.g., lead, mercury) and adverse health risks in a targeted population [25].

### Pharmacological Drug Consumption

Drug consumption is analyzed based on the “excreted mass” observed in wastewater with human excretion rates (fraction of consumed drug excreted as a form of target analyte). In this way, metabolites excreted at a larger fraction is not necessarily evaluated or equated as larger drug consumption. WBE can monitor population exposure to pharmaceuticals and drugs of abuse in communities. By measuring exposure to pharmaceuticals such as antidepressants, antipsychotics, pain relievers, and prescription opioids in wastewater, WBE can monitor their use, provide insights into the burden of disease, and “wellness” implications across residential communities [8]. WBE can also be used to assess the scope of drug use. Exposures to drugs of abuse such as opioids, stimulants (e.g., cocaine, amphetamines), THC, newer synthetic agents, and designer drugs have been characterized in wastewater, suggesting the feasibility of this modality to provide population-level data on substance use [4]. Accurate and real-time data regarding drug use, overdose, case-fatality rates, and treatment is important in guiding community efforts at combating substance use epidemics. WBE may function in two major modalities in responding to these epidemics. First, it can be used as a method to understand the prevalence of substance use. This data can be utilized to understand the effectiveness of community-based substance use disorder interventions. Second, WBE can serve as an early warning sentinel system to detect the emergence of drug use epidemics. One can monitor these in tandem with human health biomarkers (such as creatine and cortisol) which are used as controls for ebbs and flows. For example, a rise in metabolite A versus no rise in control A could be indicative of consumption changes. Of note, longitudinal WBE surveillance can help confirm the presence of disease but should not be used as the sole source of information as it requires the complement of other clinical data sources. The following sections further describe the use of WBE with these modalities.

#### Wastewater-Based Assessment of Community-Level Prevalence of Substance Use

WBE can quantify trends of drug exposure beyond existing data reporting sources which capture cases of drug use or overdose through encounters of individuals at hospitals or targeted surveys [26]. Measuring concentrations of opioid compounds and their metabolites in wastewater across large catchment zones can inform researchers regarding the status of substance use within specific communities [27]. Upstream wastewater collection enables the measurement of less stable metabolites, which has an accurate scope of drug consumption with spatial granularity. By selecting strategic upstream sampling locations, exposure and prevalent opioid use can be described for key areas of interest, while downstream analysis can be leveraged to understand population-level use [28]. This data can additionally be combined with geographic information systems (GIS) mapping to visualize varying concentrations of substance use by regional catchment areas. By measuring the presence of several commonly used opioids, public health authorities can target programs like opioid prescription takeback and community outreach for medication-assisted treatment (MAT) into areas of high opioid use [8]. This data visualization may help position emergency medical services (EMS) or peer counselors in areas with high opioid use to potential overdose. Additionally, by measuring opioid reversal agents like naloxone, insights into the prevalence of overdose and treatment can help build capacity around opioid use outreach programs and identify potential data gaps [29].

#### Wastewater-Based Sentinel Surveillance for Emergence of New Drugs

Wastewater surveillance can also be used prospectively as an outbreak monitoring system and to detect emerging illicit drug clusters [14]. By sampling wastewater in a longitudinal pattern, detection of novel drugs of abuse or changes in patterns of ongoing substance use can help toxicologists anticipate potential clinical presentations and toxidromes associated with these agents. This technique augments existing data sources like hospital admissions, emergency department visits, drug seizures, and EMS calls [6]. It is also a comprehensive and more cost- and time-effective method of determining the prevalence of novel psychoactive substances in communities [30, 31].

Novel drugs can be identified through sequencing clinical specimens and followed by the development of methods to detect and monitor these drugs in wastewater. While individual patient clinical testing remains the “gold standard” to identify a novel illicit drug, this approach is difficult to scale across large populations and implement as a routine real-time monitoring tool. In contrast, wastewater monitoring scales rapidly to entire communities, but is less able to identify novel mutations due to the complex nature of wastewater samples. Together, WBE and clinical testing can provide a more comprehensive surveillance method, wherein emerging drugs are identified via clinical screening and rapidly deployed through WBE by the development of targeted assays. In this paradigm, new variants identified in one setting can quickly become monitored across entire populations. WBE can also monitor for the reemergence of drugs which have previously established assays (e.g., phencyclidine [PCP], designer amphetamines). Additionally, WBE can detect dangerous adulterants (e.g., levamisole in cocaine) which have existing assays.

To conduct surveillance, mass spectrometry could be applied to help identify novel parent drugs of abuse and identify fragmentation fingerprints of their metabolic products. In conjunction with structural information garnered from nuclear magnetic resonance and infrared spectroscopy, these tools help to anticipate potential changes representing novel clandestine analogs that are introduced into a consumed drug supply. These techniques may be attractive adjuncts to existing drug surveillance that relies on key informant reporting, drug seizures, and syndromic surveillance among emergency departments. WBE can function as a sentinel warning system to activate public health measures prior to the widespread onset of disease and fatalities [14]. Sewage surveillance can rapidly monitor illicit drug consumption for forecasting potential outbreaks and emerging epidemics including opioids and fentanyl analogues, as well as stimulants and methamphetamine [3]. The major constituents of cannabinoids, Δ^9^-tetrahydrocannabinol (THC), and cannabidiol (CBD), and their metabolites in sewage, are also areas of epidemiological interest with legalization trends across many states [32].

## Role of the Medical Toxicologist

Advancements in WBE will require collaboration across various disciplines including analytical chemists, water and civil engineers, public health officials, epidemiologists, infectious disease specialists, microbiologists, and toxicologists [6]. Medical toxicologists can play important roles developing novel research frontiers in wastewater epidemiology as well as integration of wastewater-based data into poison centers and other public health infrastructure. As experts in understanding drug use, management of adverse drug events, and population-level environmental exposures, toxicologists can consider new research avenues that leverage the ability of wastewater analysis to provide insights into the epidemiology of substance use as well as exposures to environmental toxins.

From a policy and clinical perspective, the traditional role of the toxicologist has been to advocate for public health. Since the inception of poison control centers focused on pediatric poisoning, toxicologists have campaigned for safer medication dispensing, and child-resistant and tamper proof packaging. Throughout the COVID-19 pandemic, the intersection of health disparities and substance use disorders is another avenue for public health advocacy [33, 34]. Integration of wastewater-based substance use monitoring gives medical toxicologists another source of information as they guide public health officials on the positioning of key substance use disorder interventions including recovery counselling, mobile outreach with MAT, prescription drug take-back programs, and overdose prevention programs. Over time, longitudinal surveillance will enable toxicologists to advise policy makers on the impact of these programs on drug use and help direct resources to evolving areas of substance abuse.

Medical toxicologists may further utilize WBE to understand emerging drug trends. Similar work occurs through the European Monitoring Centre for Drugs and Drug Addiction (EMCDDA) to monitor movement of novel psychoactive substances across member countries in the European Union. In collaboration with law enforcement, community leaders and substance use disorder advocates, warning of dangerous synthetic fentanyl analogues and opioids or other psychoactive substances detected in wastewater may further prompt surveillance at hospitals, patient education, and outreach. Integrated into other early warning systems like the National Institute on Drug Abuse (NIDA) National Drug Early Warning System (NDEWS), wastewater may provide a leading trend that prompts toxicologists to consider intoxication and poisoning with emerging drugs of abuse.

## Frontiers in Research

### Addressing the Current Opioid Epidemic

WBE can provide a comprehensive and time-efficient method of determining the prevalence of synthetic opioids in communities [30]. From 2013 to 2017, the number of known opioid overdose deaths in the USA increased 90%, from 25,000 to nearly 48,000 [35]. This increase was primarily driven by increases in deaths involving illicitly manufactured fentanyl or fentanyl analogs (carfentanil, furanyl fentanyl, methoxyacetyl fentanyl) mixed with heroin or pressed into counterfeit prescription pills. Illicit fentanyl was involved in approximately two-thirds of opioid deaths during January–June 2018 [35].

Access to accurate opioid use data is essential to the management of the opioid crisis. By leveraging upstream and downstream wastewater sampling, researchers can address important questions around opioid utilization in various communities. Wastewater-based data may uncover segments of the population that do not access medical care for their opioid dependance or toxicity and therefore do not receive individual testing or treatment. Understanding the distribution of opioid consumption may help infer geographic at-risk areas which may then be targeted for intervention. Longitudinal surveillance of these areas may help investigators understand the effects of these interventions in the context of changing concentrations and therefore prevalence of opioid use. In addition to focusing interventions toward those individuals with opioid use disorder (OUD), researchers can also visualize opioid use in the context of important social determinants of health like race, income, employment, and access to healthcare. These data visualization investigations may provide an alternative geographical lens to understand the real-world impact of such factors on opioid use.

In addition to mapping and measuring prevalence of opioid use, WBE research can also provide screening analysis to uncover covert opioid use. Instead of reacting to novel toxidromes or surges of overdose deaths, screening of wastewater can be used to both anticipate and trace patterns of opioid emergence in communities. For example, untargeted high-resolution mass spectrometry (HRMS) that uncovers a clandestine opioid or synthetic fentanyl can trigger public health officials to issue alerts to individuals with OUD to mitigate overdoses. Comparisons of newly uncovered opioids can be compared to known structures in opioid assay libraries to understand potential strategies used by illicit manufacturers to develop synthetic analogs [36–38]. Discovery of these novel opioids may then be traced through wastewater catchment areas to recognize the transit of these compounds through various communities.

Current data collection methods are limited by scope and time delays and would be enhanced by larger database acquisition techniques using WBE analysis on drug consumption. For example, mass spectrometry paired with liquid chromatography or gas chromatography allows analysis of multiple opioids and their metabolites. WBE databases are now composed of numerous opioids including natural opiates (morphine and codeine), their semi-synthetic derivatives (heroin, hydromorphone, hydrocodone, oxycodone, oxymorphone, meperidine and buprenorphine), and fully synthetic opioids (fentanyl, methadone, tramadol, dextromethorphan, and propoxyphene), as well as their metabolites (6-monoacetylcodeine, dextrorphan, normorphine, and O-desmethyltramadol) [39, 40]. These databases can be monitored to detect changes to MS fingerprints to gauge whether there are amino acid/peptide additions, substitutions, or deletions. When monitoring a known synthetic opioid agent and noting a decrease in prevalence in samples over time, but seeing a rise of a similar unidentified fragment, this may represent a clue that an agent is being altered.

WBE data may serve as a complementary data source to understand the ground truth of opioid-related overdoses and deaths [27]. WBE with smart sewer selection and robotic wastewater collection is now available to detect the presence of specific opioids and intervention substances such as naloxone, methadone, and buprenorphine. These findings suggest that WBE might be used to detect patterns of opioid exposure and may ultimately provide information for opioid use disorder treatment and harm reduction programs [8]. Integration of data sources surrounding racial and ethnic barriers to access to care may also help understand the effects of these disparities on the opioid epidemic. Research to develop a novel and sustainable method for understanding the broad impact of the COVID-19 pandemic on OUD and access to MAT programs is required. Also, multimodal sentinel systems that can simultaneously detect emerging pharmaceutical and biological pathogens in wastewater, while monitoring their intertwined impact and the effects of their associated mitigation policies are being developed [8].

## Methamphetamine and Stimulant Assay Development

The opioid epidemic began as a prescription pill problem, which morphed into a widespread heroin addiction, and then a fentanyl crisis. These three overlapping events have often been commonly referred to as the “Three Waves of the U.S. Opioid Epidemic.” The latest “Fourth Wave” of the overdose epidemic began when death rates from stimulants combined with potent opioids started to rise. According to recent US death certificate data, cocaine overdoses have increased to 15,000 deaths per year and methamphetamine overdose deaths have risen to over 11,000 per year [41, 42]. Methamphetamine is currently one of the most widely used illicit drugs in Europe and is now recognized as an emerging micropollutant [43]. Amphetamine and methamphetamine are recognized contaminants in both municipal wastewater influent and effluent portals [44].

One potential application for wastewater surveillance is to measure the increasing use of methamphetamines. Combinations of stimulants (methamphetamine and cocaine) with synthetic fentanyls were initially detected through post-mortem testing of suspected drug overdoses and drug screening of individuals in emergency departments. Population level surveys demonstrate an increasing rate of methamphetamine positivity across the USA. Globally, detection of amphetamines has been described in wastewater systems in China [45, 46], Australia [47], New Zealand [48], and throughout Europe [49–51], although analysis is confounded by the prevalence of other stimulant xenobiotics that may share similar metabolic pathways. Quantifying these drugs of abuse may be more technically challenging compared to opioids given common metabolic pathways and homology between methamphetamine and other amphetamines used pharmaceutically in clinical care. Despite this, integration of methamphetamine testing in wastewater networks could serve as a method to detect the resurgence of this drug in communities. Specialized assays including the use of chiral columns that can differentiate between L and R isomers of amphetamines may help lend some specification to WBE of stimulant compounds.

In China, investigators are tracing methamphetamine and amphetamine sources via concentration and enantiomeric profiling of these two compounds from the black market to receiving wastewater sites. They found methamphetamine in wastewater predominantly arises from patterns of abuse [52]. A 7-year Australian study further demonstrated that enantiomeric profiling in wastewater-based epidemiology can provide valuable information for evaluating the origin of amphetamine in wastewater as either a metabolite of methamphetamine consumption or amphetamine itself [53]. Levels of amphetamine and methamphetamine were recently quantified at sewage treatment plants in San Diego, California. Notably, methamphetamine per-capita consumption rates in this region were found to be the highest rates ever reported for the USA or Europe confirming that distribution and use are surging in this area of California near the US-Mexico border [54].

In terms of the route of exposure, recent analysis of N, N-dimethylamphetamine in wastewater determined this to be a pyrolysis marker and synthesis impurity and indirect indicator of methamphetamine smoking. Similarly, with crack cocaine, in addition to metabolites benzoylecgonine and cocaethylene, the pyrolytic products anhydroecgonine and anhydroecgonine methyl ester are produced from the smoke of crack and are relatively stable in wastewater [55]. Like methamphetamine, cocaine use is also on the rise. In a recent WBE study from New York City, the drug group concentrations present in the wastewater samples (in decreasing order) were cocaine, nicotine, opioids, cannabis, and amphetamines. When analyzing these individual compounds and their metabolites, the highest normalized concentration was benzoylecgonine (BE), followed by cotinine, morphine, and 11-*nor*-9-carboxy-tetrahydrocannabinol (THCCOOH) [56].

### Health Implications of THC Legalization

A global systematic review and meta-analysis to estimate the rank and consumption rate of illicit drugs through WBE indicated that tetrahydrocannabinol (i.e., cannabis) had the highest consumption rates [57]. As legalization campaigns continue, cannabinoid constituents Δ9-tetrahydrocannabinol (THC) and cannabidiol (CBD), and their metabolites released into wastewater confluents, are expected to increase significantly. WBE could be an important public health monitoring tool during these societal and legal changes. WBE could be used to measure THC and other cannabis metabolites to monitor the impact of legalization on overall consumption. Combining consumption data with economic data from the highly regulated marketplace could enable estimation of the magnitude of illicit use when compared across different states with different policies. Monitoring consumption of other illicit drugs could provide insight on the impact of future legalization of these agents.

### Seasonal WBE Monitoring and Mass Gatherings

Information based on wastewater epidemiology and consumption behavior suggests that drug use monitoring may benefit from seasonal surveillance and further analysis during key calendar dates, holidays, and weekends [56, 58, 59]. There is a long-standing association between traditional and emerging drugs at warm weather mass gathering such as music festivals featuring heavy metal, rock, pop, country, folk, ethnic, dance, and trance. The occurrence of illicit drugs, their metabolites, and psychoactive compounds is a further area prime for WBE research and is currently under investigation in Europe [60]. Similar studies are needed in the USA once closely regulated mass gathering events are permitted in the post-COVID era. These mass gatherings provide a unique window to emerging drug trends in participating populations.

Research should also consider important confounding factors like contamination of catchment areas from migratory populations. For example, the presence of college students in a small town during times where classes are in session may dramatically alter the demographic and exposures discovered through wastewater analysis. Similarly, monitoring across various cities may demonstrate movement of specific drugs of abuse associated with migration of specific populations.

### Monitoring Community Mental Health and Pandemic Wellness

Numerous publications describe the detection of mental health or “wellness” medications and their metabolites in wastewater, including benzodiazepines, antidepressants, and antipsychotics [3, 61–65]. WBE can be further utilized to estimate the burden of pain treatment in a population by quantifying pharmaceutical compounds to estimate the consumption of drugs used to relieve pain, such as opioids, acetaminophen, and nonsteroidal anti-inflammatory agents [66]. One recent investigation documented the mean consumption rate of six major substance groups (antidepressants, antiepileptics, antihistamines, antihypertensives, synthetic opioids, and central nervous system stimulants) correlated with disparities in household income, marital status, and age of the contributing populations. The detection frequency of (SARS-CoV-2) RNA in wastewater and COVID-19 test positivity in the studied sewer-shed areas was also determined. Overall, this study demonstrated the utility of WBE for assessing the population-level substance use patterns during a public health crisis such as COVID-19 [67]. Like monitoring for COVID-19 disease, a nationwide system of wastewater geospatial maps could be generated to demonstrate patterns of substance use across regions. These maps could reveal community vulnerabilities or susceptibilities as a function of race, ethnicity, socioeconomic status, occupation, age distribution, climate, and access to healthcare [6].

### WBE in Global Low-Resource Settings

WBE has potential in low-to-middle income countries to inform drug monitoring and prevention strategies in regions where substance abuse data may be limited due to funding constraints and lack of government structures to facilitate conventional monitoring [68]. Substance use disorders tend to be prevalent in lower-income populations. Selected populations are also more likely to be living near industrialized areas exposing them to unregulated chemicals and compounds. WBE surveillance may bring about policy change reflecting these various social determinants of health. Low-income countries may not have the necessary finances and sewer system infrastructure to support WBE as in the USA and Europe. However, this remains an important avenue for future funding and research. In these low-resource settings, WBE is potentially more cost-effective than widespread individual testing. A WBE study included the quantification of several drugs of abuse in raw wastewater samples and WWTPs in Africa [69, 70]. Non-racemic 3,4-methylenedioxymethamphetamine (MDMA) and methamphetamine, as well as the metabolite of cocaine, benzoylecgonine and heroin, O-6-monoacetylmorphine (O-6-MAM) were detected, paving the way for future research and public health interventions in these resource-challenged settings. In addition, global monitoring of antimicrobial resistance surveillance is another contribution of WBE [71].

### Biomarkers and Biosensors

WBE provides technical support for the detection of pharmaceutical trends and wastewater contaminants and the development of early warning sensing systems [72]. WBE’s usefulness could be expanded by targeting endogenous biomarkers (metabolites) that are elevated in sewage following pathogen or drug exposures. Benefits of targeting these indirect markers include reduced analytical costs and broader immunoassay availability to serve as early indicators of surges and dynamic drug trends [6].

There is an additional need to develop portable assays that enable analysis at the site of sample collection. Biosensors have emerged as effective alternatives that can test sewage in the field [73]. Biosensors are highly sensitive and selective tools for the analysis of sewage biomarkers due to their ease-of-use, low cost, and rapid analysis. [2] These small devices utilize biochemical reactions mediated by a biological receptor recognition element, such as nucleic acids, antibodies, enzymes, and microorganisms, to detect targets based on optical, electrical, and thermal signals [74]. Traditional collection methods requiring on-site data acquisition are labor intensive. Biosensors augment wastewater sampling by allowing remote real-time monitoring. As a result, this technology may decrease the need for in-person testing and sampling. Additionally, these “smart” sewer systems monitor and alarm when certain immunoassay threshold levels are reached (e.g., mercury and lead contamination). Future use of the biosensor technology for WBE will enable on-site monitoring of sewage, which will provide data for public health assessment and timely intervention. Biosensors have already played an important role in quantification of pharmaceuticals in wastewater for assessment of drug consumption in selected populations [3].

### Potential WBE Research Funding Sources

Given the wide range of application for WBE as an adjunct to existing pharmacovigilance strategies, multiple potential funding sources exist. It will be important for researchers to match the priorities of these sources with the capabilities of WBE. While there are many avenues to develop and deploy WBE monitoring, integrating this data source into existing data streams that are used to monitor population health is also critical. The ability to build long-lasting, permanent capacity for the use of WBE can lead to sustained evaluation of wastewater as a key source of health data.

Potential funding sources for wastewater-based epidemiological research and harm reduction interventions include the National Institutes of Health (NIH), The National Institute on Drug Abuse (NIDA), The Health Resources and Services Administration (HRSA), Center for Disease Control and Prevention (CDC), Agency for Toxic Substances and Disease Registry (ATSDR), industry grants, pharmaceutical companies, small business ventures, and nongovernmental foundations grants (Table 1).

**Table 1:** Funding sources and priorities for WBE research.

Funding source	Topical interest
National Institutes of Health / NIDA	WBE research implications in specific disease states Development of novel assays Monitoring drug use trends and consequences
HRSA	WBE monitoring of geographically isolated, economic or medically vulnerable populations
CDC/NIOSH	Workplace safety and health WBE surveillance of national, regional, state, community-level wastewater contaminants
ATSDR	Monitoring of harmful health effects from exposure to hazardous substances and chemicals (e.g., pesticides, heavy metals)
Foundation grants	WBE for environmental health monitoring and wastewater technology advances in LMICs

*LMICs* low-to-middle-income countries

## Pitfalls and Limitations

### Wastewater Dilution Effect and Metabolite Stability

Urinary and fecal information in wastewater is more difficult to analyze as compared to clinical specimens. Biological and pharmacological information in wastewater is often diluted and may be compromised by the wastewater matrix that includes graywater from household and industrial facilities and by the presence of biofilm in sewer pipes [75–77]. The mixed content is relatively stable, although diminished from the action of biological processes [53]. The continual transformation of parent analytes that are targeted by monitoring efforts further complicates the modeling of loadings of analytes that originate from excreta [78]. A major challenge is the requirement for much lower limits of analytical detection in the presence of background content, in addition to detection capability in already diluted samples. There is also a need to normalize targeted analyte levels to the size of the contributing population so that values can be standardized and compared with a “gold standard” [71].

We acknowledge that selected parent compounds and metabolites are suitable for downstream WBE. In addition, chemical degradation in sewers may affect parent/metabolite ratios (e.g., THC-COOH is reconverted to THC-OH or THC, or a glucuronidated compound is cleaved to an upstream metabolite or parent). Therefore, upstream sampling is preferred to capture these more unstable metabolites. Correction factors to account for variations in sewer networks and wastewater matrices are ways to mitigate these limitations (e.g., size of sewer network, access point, dilution, temperature, and degradation of metabolites). Admittedly, certain substances and analytes that have site-/matrix-specific degradation may not be suitable for WBE. Further research is needed to confirm suitability of various substrates and analytes for WBE before interpreting these data.

### Operational Laboratory Requirements

The equipment needed to analyze sewage are costly instruments that are fixed in specialized laboratories and operated by highly trained technicians. These include liquid chromatography-tandem mass spectrometry (LC–MS/MS) and gas chromatography mass spectrometry (GC–MS) for the detection of small organic chemicals and their metabolites, and polymerase chain reaction (PCR) for the detection of nucleic acids and genetic information for biological pathogens [73]. Additionally, due to the sordid nature of wastewater samples, timely, costly, and methodical sample preparations such as solid-phase extraction (SPE) are needed prior to analysis for sample cleanup. These laboratory requirements limit the broad applications of several WBE techniques [3] paving the way for real-time, lower cost, portable devices (such as biosensors) with direct field research applications.

### Surveillance, Population Data

Although wastewater-based surveillance data is de-identified, inclusive, and unbiased, it could add to increased surveillance scrutiny already faced by certain groups, if not designed explicitly to avoid these issues [10]. With sewage epidemiology monitoring drugs of abuse, one form of resistance is community leaders declining monitoring because they do not want to be perceived as a potential “hotspot” for illicit drug use [6]. There are also privacy concerns and the risk of stigmatization with public health interventions such as harm reduction programs when considering the social implications of wastewater surveillance. Therefore, these surveillance programs should be developed to address both anonymity and inequality concerns. In response, upstream sampling can be designed to collect aggregate but not household-level data as to not target demographics consistent with existing socioeconomic variability. Otherwise, these populations may be subject to over-surveillance, profiling, and restriction policies [10].

## Conclusions

New frontiers in research and sewer system surveillance include opioid MAT program assessments, pharmaceutical-related community wellness, and harm reduction amid the COVID pandemic. Monitoring emerging drug trends (e.g., fentanyl analogues) and resurging stimulants (e.g., methamphetamine and cocaine) is another avenue for future investigation. Further developments of assay libraries for monitoring substance use trends in selected populations with pharmaceutical surveillance are needed. Innovations in WBE featuring biomarker assays and biosensors represent the latest cutting-edge contributions to “smart” sewer systems. Toxicologists can play a vital role in wastewater surveillance and analysis while collaborating with experts in public health, infectious disease, and water engineering to further develop wastewater monitoring, research, and innovation.
